# Southern Medical College Announcement

**Published:** 1879-07-20

**Authors:** R. C Word

**Affiliations:** Dean pro tem., Atlanta, Georgia


### THE SOUTHERN MEDICAL COLLEGE.

Faculty:

A. S. PAYNE, M.D.,
Professor of Theory and Practice of Medicine.

Professor of the Principles and Practice of Surgery. (To be filled.)

T. S. POWELL, M.D.,
Professor of Obstetrics and Diseases of Women, and Lecturer on Medical Ethics.

R. C. WORD, M.D.,
Professor of Physiology.

G. M. McDOWELL, M.D.,
Professor of Materia Medica and Therapeutics.

Professor of Chemistry. (To be filled.)

WM. PERRIN NICHOLSON, M.D.,
Professor of General and Pathological Anatomy.

W. T. GOLDSMITH, M.D.,
Professor of Diseases of Children, and Lecturer on Clinical Gynæcology.

H. F. SCOTT, M.D.,
Professor of Medical and Surgical Diseases of the Eye and Ear.

G. G. CRAWFORD, M.D.,
Professor of Operative and Clinical Surgery.

LINDSAY JOHNSON, M.D.,
Demonstrator of Anatomy.

Auxiliary Professors:

J. F. ALEXANDER, M.D., Auxiliary Professor of Practice of Medicine, and Lecturer on Clinical Medicine.

W. G. OWEN, M.D., Auxiliary Professor of Physiology and Lecturer on Diseases of the Nervous System.

G. G. ROY, M.D., Auxiliary Professor of Materia Medica and Lecturer on Therapeutics and Medical Jurisprudence.

H. B. LEE, M.D., Auxiliary Professor of Obstetrics and Diseases of Women.

J. C. OLMSTEAD, M.D., Lecturer on the Genito-Urinary Organs and Venerial Diseases.

LINDSAY JOHNSON, M.D., Auxiliary Professor of Surgery, Lecturer on Minor Surgery, and Instructor on Splints and Bandages.

A. J. PINSON, M.D., Assistant to the Lecturer on Minor Surgery.

### Announcement of the Southern Medical College.

The Board of Trustees and Faculty of the Southern Medical College are gratified to announce to medical students, to the medical profession, and the public generally, that this new Institution has been successfully established, and that the First Course of Lectures will commence in Atlanta, Ga., on the second Monday of October, 1879.

The central position of Atlanta; its accessibility; its favorable location as a financial and commercial centre, together with its extraordinary healthfulness and exemption from devastating epidemics to mar its prosperity or disturb its enterprises; all combine, in a peculiar degree, to proclaim its adaptability and fitness as the great medical centre of the South.

The *College Building*, now in process of erection, will be commodious, convenient and elegant. Its position is central, being within two hundred yards of the Union Passenger Depot, and nearer still to the leading hotels and a number of good boarding houses. It is also equally convenient to the centre of the great street railroad system of Atlanta, which radiates to every part of the city.

The *Lecture Rooms* will be large and well ventilated, and every apartment furnished with water from the water-works, lighted with gas, and supplied with all the modern conveniences and improvements.

The *Dissecting Room* will be in the third story of the building, thoroughly ventilated, lighted with gas, and well supplied with water.

The Museum.—The hall for the Museum will be 30x60 feet in size. It will be supplied with anatomical preparations, relics, curiosities, and specimens of every kind calculated to illustrate science and facilitate instruction in the various departments of study.

A *Library* of medical and scientific works will also be provided, and will contain all the leading medical journals of the country, to all of which the students will have access.

The *Chemical Laboratory* will be full and complete, and all necessary apparatus and material will be supplied for thorough instruction in all departments.

The Faculty elected is composed of eminent men in the profession, distinguished either as teachers, writers or lecturers, and prepared to instruct students in all the late advances in medical science. A number of experienced and intelligent gentlemen have also been elected as Auxiliary Professors and Lecturers upon special subjects. The student is thus afforded more extensive and superior facilities for the acquirement of a thorough and practical medical education.

CLINICAL INSTRUCTION.—While we cannot as yet boast of large hospitals in Atlanta, we have an abundant supply of clinical material in the city. Daily clinical lectures will be given—cases exhibited and treated before the class. The Faculty and Auxiliary Professors and Lecturers will spare no pains to furnish the students with ample clinical and practical instruction.

PRACTICAL ANATOMY.—Dissections will be done at night. The Demonstrator and his assistants will give attention to this department, and ample material and every facility will be afforded the students for instruction in this important branch.

| | |
|---|---|
| FEES.—Matriculation (to be taken once) | $ 5 00 |
| Tickets of Full Course | 50 00 |
| Demonstrator's Fee (per term) | 10 00 |
| Clinical Instruction—no charge. | |
| Diploma Fee | 30 00 |

All fees, except for diploma, must be paid at the beginning of the session, unless satisfactory arrangement be made with the Dean for future payment.

*Graduates of other Medical Schools*, recognized by this Institution, will be admitted to the lectures by paying the matriculation fee, unless they apply for graduation, in which case the diploma fee will be required.

Students who have attended and paid for two full courses of lectures in this Institution, will be allowed to attend a third or fourth course of lectures without charge, and the Faculty recommend them to avail themselves of this privilege in all cases where their pecuniary and other circumstances will admit.

A student who attended his first course in another school and his second in this school, and desires to attend a third course before applying for graduation, may do so by paying one-half of the ordinary fees.

Students may attend the lectures of one or more professors by paying the matriculation fee and purchasing single tickets at $10 each.

BENEFICIARIES.—The charter of the Southern Medical College does not require the admission of beneficiaries into the Institution. Yet the Trustees and Faculty, recognizing the claims of poor, intelligent and deserving young men of our country, will admit a limited number of privileged students of this class on the payment of the matriculation fee of $5.00 and the Demonstrator's fee of $10.00. Students of this class will be known only to the Dean, and will be granted all the privileges of the Institution.

REQUIREMENTS FOR THE DEGREE OF DOCTOR OF MEDICINE.—

1. The candidate must be of good character, and must have attended two full courses of lectures, the last of which shall be in this Institution.
2. He must have attended the Dissection in this school while a student thereof.
3. He shall undergo a personal and satisfactory examination before the Faculty.
4. He must write a thesis composed by himself on a medical topic, or make a clinical report in some department of medicine. This thesis or report to be handed to the Dean one month before the close of the session, accompanied with the diploma fee. In case of failure to pass a satisfactory examination, the diploma fee will be refunded.

TEXT BOOKS RECOMMENDED.—

*Anatomy*—Grey, Wilson, Richardson, Leidy's Human Anatomy, Sharpey & Quain.
*Surgery*—Gross, Erichsen, Ashurst.
*Practice*—Flint, Roberts, Niemeyer, Watson & Atkin.
*Physiology*—Carpenter, Dalton, Flint.
*Obstetrics*—Leishman, Cazaux, Playfair, Ramsbotham.
*Diseases of Women*—Thomas, Barnes.
*Diseases of the Eye, Ear and Throat*—Wells, Stillway, Jones.
*Diseases of Children*——Smith, West, Meigs & Pepper.
*Chemistry*—Fownes, Roscoe, Attfield, Barker.
*Materia Medica*—Biddle, Stille, Wood, U. S. Dispensatory.
*Dermatology*—Duhring, Fox, Wilson.
*Nervous Diseases and Clinical Medicine*—Hammond, Trousseau.
*Medical Jurisprudence*—Stille, Wharton, Taylor, Beck.
*Urinary Diseases*—Roberts, Thompson, Gross.

BOARDING.—Good board may be had at $4 to $6 per week. Students on arriving in the city should at once call on the Janitor or Dean at the College building.

For further information, address

R. C WORD, M.D.,

*Dean pro tem., Atlanta, Georgia.*

N. B.—The Faculty and Board of Trustees appeal to students, to medical men, and to all others desiring to aid in a good work, to donate books, relics, curiosities, anatomical preparations, and anything adapted to the College Museum and Library. The Southern Express Company has generously consented to send all articles of five pounds and under free of freight for this purpose. Heavy articles may in some cases be so divided as to give us the benefit of this free transportation, and when practicable our friends are requested to do this. Otherwise the freight will be paid at this terminus.

The names of the donors will be inscribed upon each specimen, with the date and his place of residence, which will ever attest his generosity and prove an object of perpetual interest and usefulness to future generations.

Articles donated should be marked "For Southern Medical College," and addressed to Dr. R. C. Word, Secretary of Board.

☞The above announcement will soon be issued and distributed in pamphlet from, with possibly some slight changes and blanks filled out.

